# Kinetic modelling: an integrated approach to analyze enzyme activity assays

**DOI:** 10.1186/s13007-017-0218-y

**Published:** 2017-08-25

**Authors:** Jelena Boeckx, Maarten Hertog, Annemie Geeraerd, Bart Nicolai

**Affiliations:** 1 BIOSYST-MeBioS, KU Leuven, Willem de Croylaan 42, 3001 Louvain, Belgium; 2 Flanders Centre of Postharvest Technology, Willem de Croylaan 42, 3001 Louvain, Belgium

**Keywords:** Kinetic model, Enzyme activity, Alcohol dehydrogenase, Pyruvate decarboxylase, Spectrophotometric enzyme assay

## Abstract

**Background:**

In general, enzyme activity is estimated from spectrophotometric data, by taking the slope of the linear part of the progress curve describing the rate of change in the substrate or product monitored. As long as the substrate concentrations are sufficiently high to saturate the enzyme and, the velocity of the catalyzed reaction is directly proportional to the enzyme concentration. Under these premises, this velocity can be taken as a measure of the amount of active enzyme present. Estimation of the enzyme activity through linear regression of the data should only be applied when linearity is true, which is often not the case or has not been checked.

**Results:**

In this paper, we propose a more elaborate method, based on a kinetic modelling approach, to estimate the in vitro specific enzyme activity from spectrophotometric assay data. As a case study, kinetic models were developed to estimate the activity of the enzymes pyruvate decarboxylase and alcohol dehydrogenase extracted from ‘Jonagold’ apple (*Malus x domestica* Borkh. cv. ‘Jonagold’). The models are based on Michaelis–Menten and first order kinetics, which describe the reaction mechanism catalyzed by the enzymes.

**Conclusions:**

In contrast to the linear regression approach, the models can be used to estimate the enzyme activity regardless of whether linearity is achieved since they integrally take into account the complete progress curve. The use of kinetic models to estimate the enzyme activity can be applied to all other enzymes as long as the underlying reaction mechanism is known. The kinetic models can also be used as a tool to optimize the enzyme assays by systematically studying the effect of the various design parameters.

**Electronic supplementary material:**

The online version of this article (doi:10.1186/s13007-017-0218-y) contains supplementary material, which is available to authorized users.

## Background

The most diverse and largest group of proteins are enzymes, biological catalysts enhancing the rates of metabolic reactions and thereby play an important role in the regulation of metabolic steps within a cell [1]. Michaelis and Menten discovered that the activity of an enzyme is highly dependent on conditions such as temperature, pH and the availability of substrate and cofactors inside the cell [2].

Many assays are described to measure enzyme activity in vitro [3, 4]. Generally, data obtained with enzyme assays can only be reliably compared between different experiments and between different labs and publications if each time conditions that guarantee the highest possible enzyme activity have been used. Therefore, temperature, pH, nature and strength of ions, and the proper concentration of all assay components have to be strictly controlled [5, 6]. Most enzymatic assays are based on measuring the rate of the reaction catalyzed by the enzyme. To this end, the consumption of the substrate or the generation of a product has to be monitored over a given time. The progress of the reaction can be monitored continuously (continuous assay) using spectroscopic [7–9] or electrochemical techniques revealing the full progress curve. The advantage of a continuous assay is that the result is immediately available, and any erroneous influences and artefacts can be detected. However, if continuous monitoring is not feasible one has to obtain data at limited discrete time points using the same techniques or stop the reaction and measure the total amount of product formed or substrate consumed within the given reaction time by a subsequent chemical indicator reaction or a separation method (stopped assay) [4–6].

As long as the substrate concentrations are sufficiently high to saturate the enzyme and, thus ensure operation at its maximum rate, the velocity of the catalyzed reaction is directly proportional to the enzyme concentration. Under these premises, this velocity can be taken as a measure of the amount of active enzyme present. However, to experimentally realize substrate saturation and engage 99% of the enzymes binding sites, the substrate is required to be present in a 100-fold surplus of the K_m_ value [5]. The enzyme database BRENDA contains a wide range of K_m_ values for many enzymes and their substrates. As these K_m_ values differ widely not only between species but also within one species, it becomes difficult to know when substrate saturation is achieved. Moreover, enzyme saturation cannot always be maintained throughout the course of the reaction due to substrate conversion; as the reaction progresses and substrate gets depleted, the reaction slows down and the progress curve becomes non-linear. While this final non-linear phase provides valuable information on the kinetics of the underlying reaction, it is the initial linear part of the progress curve that provides a proper measure of the maximum enzyme activity realized under the imposed assay conditions. Assuming linearity, linear regression can be applied to this initial part of the progress curve to calculate the rate of change as a measure of the enzyme activity and, therefore, of the amount of active enzyme present. However, the assumption of linearity is, too often, not explicitly checked in a stopped assay. Only when the reaction time of the stopped assay is restricted to the linear part of the progress curve, proper results will be obtained [4–6].

This paper aims to promote an alternative, more elaborate procedure to estimate the in vitro specific enzyme activity from the progress curve based on a kinetic modeling approach taking into account the complete progress curve, including the non-linear range. The kinetic models are to be dedicated to the enzyme activity studied. In contrast to the linear regression approach, the kinetic modelling approach is also valid when the assumption of linearity is not fulfilled. Furthermore, the kinetic models are able to estimate the maximum enzyme activity, whether or not optimal assay conditions were achieved.

The proposed approach is demonstrated on the determination of the in vitro specific enzyme activity of two fermentation enzymes, pyruvate decarboxylase (PDC) and alcohol dehydrogenase (ADH), extracted from Jonagold apple (*Malus x domestica* Borkh., cv. ‘Jonagold’). To prolong their storage life and maintain the quality of the fruit, Pome fruit are commonly stored at low temperature in combination with low O_2_ and/or high CO_2_ partial pressure [10]. However, the optimal gas composition is critical as the respiratory metabolism at low oxygen conditions can shift to a fermentative metabolism. This shift is expected to be regulated by changes in the level of involved enzyme, which affect their overall activity [11, 12]. Kinetic models for pyruvate decarboxylase and alcohol dehydrogenase were developed based on Michaelis–Menten and first order kinetics.

## Methods

### Plant material

‘Jonagold’ apples (*Malus* x *domestica* Borkh., cv. ‘Jonagold’) were harvested from an orchard in Rotselaar, Belgium on September 24 2015, during the commercial harvest window for long term storage of ‘Jonagold’ apples.

### Chemicals

MES hydrate, triton X-100 (laboratory grade), acetaldehyde (ACS reagent, ≥99.5%), β-nicotinamide adenine dinucleotide [reduced disodium salt hydrate ≥97% (HPLC)], thiamine pyrophosphate, alcohol dehydrogenase (*Deinococcus radiodurans* recombinant from *E. coli* ≥10,000 units mL^−1^), sodium pyruvate and polyvinylpyrrolidone were purchased from Sigma-Aldrich (Overijse, Belgium). Dithiothreitol was obtained from VWR International (Leuven, Belgium) and magnesium chloride from Chem-Lab Analytical (Zedelgem, Belgium).

### Enzyme extraction

Enzymes were extracted according to Saquet and Streif [13], with the following modifications. For each replicate, 0.5 g frozen apple tissue was homogenized with 1 mL ice-cold extraction buffer. The extraction buffer contained 100 mM 2-(*N*-morpholino) ethane sulfonic acid (MES) buffer (pH 7.5), 5 mM dithiothreitol and 2.5% (w/v) polyvinylpyrrolidone and 0.02% (w/v) triton X-100. The homogenate was kept below 4 °C for 20 min and stirred continuously. The homogenate was centrifuged at 14,000*g* for 20 min at 4 °C. In order to achieve 80% efficiency for the extraction, the pellet was extracted once more using the same protocol. The supernatants of both extractions were well mixed and retained as enzyme extract for measuring PDC and ADH activities. The protein concentration of the crude extracts was determined using the Bradford assay [14]. The measured enzyme activity in this study was expressed relative to the total protein content of the sample.

### Enzyme activity measurement

The enzyme activity of pyruvate decarboxylase (PDC) and alcohol dehydrogenase (ADH) was assayed as described by Ke et al. [10], Imahori et al. [15] and Saquet and Streif [13], with the following modifications. In order to study the in vitro enzyme activity of PDC and ADH, the oxidation of NADH by the extracts was monitored spectrophotometrically. ADH activity was measured by mixing 100 µL of crude extract with 115 µL 1 M MES buffer (pH 6.5), 25 µL of 800 mM acetaldehyde and 10 µL of 10 mM NADH. PDC activity was assayed through coupling with the ADH catalyzed reaction. To this end, 100 µL crude extract was mixed with 90 µL of 1 M MES buffer (pH 6.5), 10 µL of 5 mM thiamine pyrophosphate, 10 µL of magnesium chloride, 5 µL of commercial ADH solution (containing 50 enzyme units), 25 µL of 500 mM sodium pyruvate and 10 µL of 10 mM NADH. In this PDC assay, a surplus of commercial ADH was added to make sure that acetaldehyde formed through the PDC reaction was directly converted to ethanol, meanwhile oxidizing NADH. For both assays the reactions were initiated by addition of the reaction buffer, which contained both substrates (NADH and pyruvate/acetaldehyde) and cofactors. The oxidation of NADH was measured by continuously recording the decrease in absorbance at 360 nm using a spectrophotometer (Multiskan spectrum microplate spectrum, ThermoLabsystems, Finland) until a steady base level was reached. To ensure the noise in the reaction was limited to a minimum, the absorbance spectra of all compounds involved in the reaction mixture were measured between 200 and 400 nm. The signal to noise ratio was determined by dividing the spectra of NADH by the sum of the spectra of the other compounds involved in the reaction. In order to check the technical and biological variation between the different extracts, absorbance of different extracts was measured at 360 nm in duplicate. All assays and additional spectrophotometric measurements were executed in flat bottom 96 well microtiter plates (Thermo Fisher Scientific) with a final reaction volume of 250 µL per well. The pH and salt concentration used in this paper was based on the existing protocols as described by Ke et al. [10], Imahori et al. [15] and Saquet and Streif [13] and not subject to further optimization.

### Determination of the absorption coefficient of NADH

The absorbance coefficient of NADH was determined by making a calibration curve with varying amounts of NADH (0–0.2–0.4–0.6–0.8–1 mM) prepared under assay conditions. Based on this calibration curve, linearity of the absorbance data measured with the spectrophotometer was confirmed up to a value of 1.887.

### Data analysis

The differential equations of the developed model (as will be described below) were implemented and parameters were estimated in OptiPa [16], a dedicated optimization tool which was developed using Matlab (The MathWorks, Inc., Natick, MA, USA). The differential equations were solved using the MATLAB solver ode45 and the model parameters were estimated using non-linear least squares optimization applying the Levenberg–Marquardt method.

## Results and discussion

### Optimization of the enzymatic assays

**Fig. 1:**
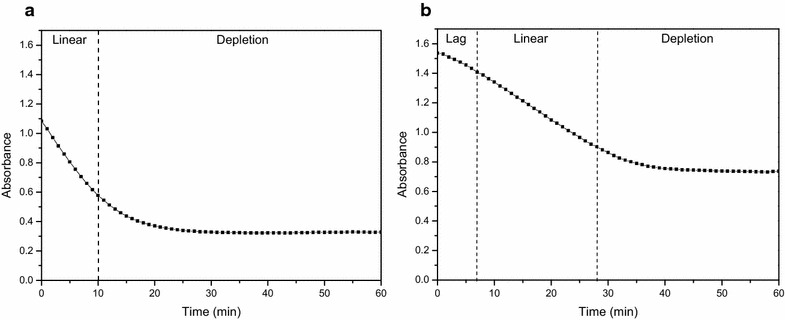
Representative example of spectrophotometric data obtained in the enzymatic assays. Spectrophotometric data obtained in the enzymatic assay to measure the activity of alcohol dehydrogenase (**a**) and pyruvate decarboxylase (**b**). Measured values (*dots*) are shown

In most enzymatic assays, enzyme activity is calculated by measuring the rate of consumption of a substrate or the production of a product over a given time period. In the case of PDC and ADH, the rate of oxidation of NADH is monitored. Given NADH has a known maximum light absorbance at 340 nm, most enzymatic assays, which depend on the conversion of NADH to NAD^+^, base themselves on the absorbance values measured at this wavelength. However, the spectrophotometric data obtained in the enzymatic assays of both PDC and ADH were not reaching zero (Fig. 1) indicating NADH may not be the only compound in the reaction mixture absorbing UV light at 340 nm, introducing unwanted noise.

**Fig. 2:**
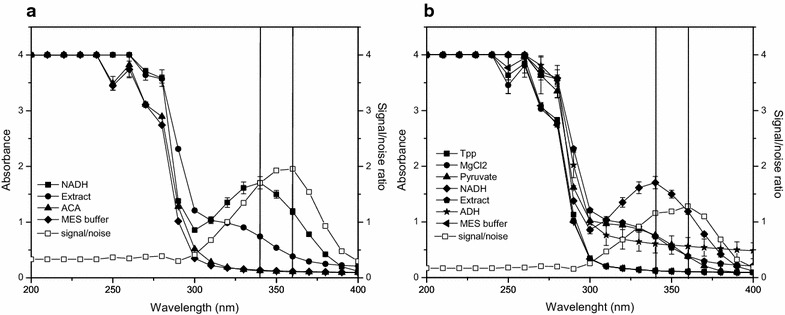
Spectra of all the compounds in the reaction mixture involved in the enzymatic assay. Spectra of all the compounds in the reaction mixture involved in the enzymatic assay to measure the activity of alcohol dehydrogenase (**a**) and pyruvate decarboxylase (**b**). *Tpp* thiamine pyrophosphate, *MgCl2* magnesium chloride, *ADH* alcohol dehydrogenase, *ACA* acetaldehyde. *Bars on top* indicate standard error (n = 3)

In general, the intensity of the signal produced by the reaction must exceed the noise produced by the other compounds by at least a factor two [5]. To understand which other compounds were absorbing UV light at 340 nm and if the noise could be limited to a minimum, the absorbance spectra of all compounds involved in the reaction mixture were measured between 200 and 400 nm (Fig. 2).

**Fig. 3:**
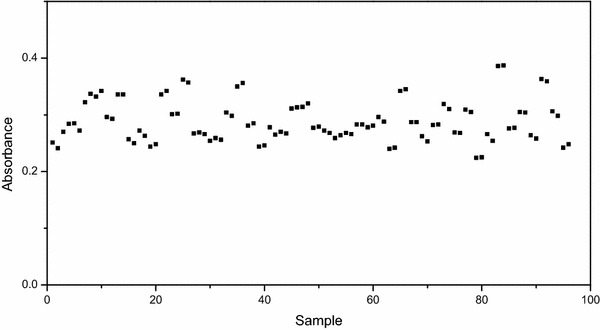
Absorbance data of 48 different extracts measured at 360 nm. Each two consecutive data points were technical repetitions of the same extract, each set of technical replicates was obtained from a different biological sample. Measured (*dots*) values are shown

Acetaldehyde showed no absorbance in the range of 320–400 nm, making NADH the only substrate of the ADH assay absorbing light in this wavelength range. However, of the substrates involved in the PDC assay, pyruvate showed a clear absorbance in the UV range. Since pyruvate is present in excess, its concentration during the enzymatic assay will stay nearly constant, causing a nearly constant absorbance signal throughout the assay, explaining the much higher base level in the PDC assay (Fig. 1b). Consequently, the decrease in absorbance in the PDC assay can still be assigned to the change in NADH. Interesting to notice is that in both example spectra the order of magnitude of the difference in absorbance between the enzymatic extracts was quite large (Fig. 2). By comparing technical and biological replicates (Fig. 3), it became clear that the variance in signal was not due to pipetting errors possibly induced by the foamy properties of the extract but rather came from compositional variation between the extracts.

In order to increase the signal to noise ratio as much as possible, the measurement wavelength for the enzymatic assays was switched from 340 to 360 nm (Fig. 2). In general, enzyme activity can be estimated from the spectrophotometric data, by taking the slope of the linear part of the progress curve resembling the rate of change in the substrate or product monitored. The concentration of the substrate, in this case NADH, can be calculated from the signal intensity applying an absorption coefficient [5]. The absorption coefficient of NADH (2.519 mM^−1^) was determined by making a calibration curve with varying amounts of NADH prepared under assay conditions, at the same time confirming the linearity of the absorbance reading up to a value of 1.887.

**Fig. 4:**
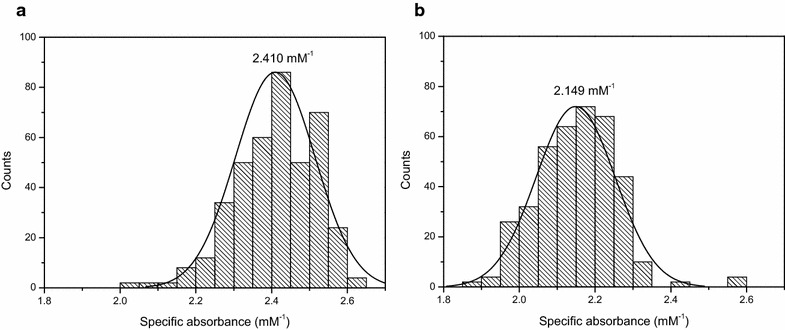
Distribution of the specific absorbance of NADH in the ADH assay (**a**) and the PDC assay (**b**). The specific absorbance of NADH is determined by the difference in absorption between the start and the end of the assay divided by the starting concentration of NADH. The average of the specific absorbance is indicated above the *graph*. The histograms are based on a total of 404 and 384 samples analyzed for respectively the ADH and PDC assay

The overall change in absorbance during the enzyme assay, is due to the conversion of NADH to NAD^+^. Assuming a complete conversion of NADH, this change in absorbance is directly related to the initial amount of NADH through its absorption coefficient. Interesting to notice is that the experimentally determined absorption coefficient of NADH (2.519 mM^−1^) differed from the absorption coefficient calculated from the average difference in absorbance between the start and the end of the enzyme assay. Additionally, this also differed between the two assays (2.410 mM^−1^ for the ADH assay and 2.149 mM^−1^ for the PDC assay, Fig. 4). This discrepancy was more pronounced for the PDC assay, as compared to the ADH assay, suggesting some matrix effects occurred in this assay. Given the enzyme extracts used between the two assays were identical, this effect was not related to the sample matrix as such but to the other assay conditions.

Estimation of the enzyme activity through linear regression of the data should only be applied when linearity is true, which is often not the case or has not been checked. For example, the spectrophotometric data of coupled reactions like the PDC assay initially showed a lag phase before reaching a linear phase (Fig. 1b). Since the enzyme activity of PDC can only be correctly estimated by taking the slope of the linear part of the progress curve, a large part of the progress curve cannot be taken into account. These data emphasize the importance of a continuous assay, as it is impossible to notice the initial non-linear part of the curve in a stopped assay. Furthermore, data like shown in Fig. 1b are pointing out the need for a more elaborate model based approach to determine the in vitro specific enzyme activity, which is also valid when the assumption of linearity is not fulfilled. To this end, dedicated kinetic models were developed.

### Kinetic model development

Kinetic models were developed to estimate the in vitro specific enzyme activity of PDC and ADH from the spectrophotometric data. The kinetic models were based on the underlying reaction mechanism of the fermentative metabolism, summarized by Eqs. 1 and 2.

1 $$ {\text{Pyruvate}} + {\text{PDC}}\underset{{k_{ - 1} }}{\overset{{k_{1} }}{\longleftrightarrow}}{\text{AC}}_{1} \mathop{\longrightarrow}\limits^{{\mathop k\nolimits_{PDC} }}{\text{acetaldehyde}} + {\text{PDC}} $$

2 $$ {\text{Acetaldehyde}} + {\text{NADH}} +
{\text{ADH}}\underset{{k_{ - 2} }}{\overset{{k_{2}
}}{\longleftrightarrow}}{\text{AC}}_{2}
\xrightarrow{k_{ADH}}{\text{ethanol}} + {\text{NAD}}^{ + } + {\text{ADH}} $$

where AC_1_ and AC_2_ are the intermediate enzyme–substrate complexes formed in the reaction.

During fermentation acetaldehyde is produced through pyruvate decarboxylation by PDC (Eq. 1). In the next step acetaldehyde is converted to ethanol by ADH using NADH (Eq. 2).

### Kinetic model for alcohol dehydrogenase

The kinetic model to estimate the activity of ADH is based on Michaelis–Menten kinetics describing Eq. 2 of the fermentative metabolism. Michaelis–Menten kinetics was used with regard to NADH only as the acetaldehyde concentration in this reaction was kept at high levels assuming saturation. The Michaelis–Menten equation to describe the conversion of NADH to NAD^+^ over time was defined as:

3 $$ \frac{{d[{\text{NADH}}]}}{dt} = \frac{{ - V_{m,ADH} \cdot [{\text{NADH}}]}}{{K_{m,ADH} + [{\text{NADH}}]}} $$

where K_m,ADH_ is the Michaelis–Menten constant and V_m,ADH_ is the maximum reaction rate which, on its turn, was defined as:

4 $$ V_{m,ADH} = k_{ADH} \cdot [{\text{ADH}}]_{0} $$

where k_*ADH*_ is the maximum number of substrate molecules converted to product per enzyme molecule per second and [ADH]_0_ is the initial concentration of ADH present in the reaction mixture. The concentration of enzyme present in the reaction mixture is related to the concentration of enzyme present in the extract ([ADH_extr_]), the volume of extract taken (*V*_*extr*_) and the total volume of the reaction mixture (*V*) leading to:

5 $$ V_{m,ADH} = k_{ADH} \cdot \left( {\frac{{\left[ {{\text{ADH}}_{\text{extr}} } \right] \cdot V_{extr} }}{V}} \right) $$

Given the theoretical rate constant k_*ADH*_ and the extract depending enzyme concentration [ADH_extr_] are two unknowns appearing as a single product term, together being responsible for the overall perceived enzyme activity, a single composite replacement term, $k_{\text{ADH}}^{\prime }$, was defined representing the ADH activity extracted from the sample. The Michaelis–Menten equation to describe the conversion of NADH to NAD^+^ (Eq. 3) was thus redefined as:

6 $$ \frac{{d[{\text{NADH}}]}}{dt} = \frac{{ - k_{ADH}^{'} \cdot \left( {V_{extr} /V} \right) \cdot [{\text{NADH}}]}}{{K_{m,ADH} + [{\text{NADH}}]}} $$

The numerical solution of this differential equation describes the concentration of NADH for every point in time ([NADH](t)), with [NADH]_0_ as the initial condition. As the absorbance measured in the enzymatic assay is the sum of the absorbance by the changing levels of NADH and the constant, but sample specific, absorbance by the extract (*A*_*extr*_), the modelled absorbance *A*_mod_ is given by:

7 $$ A_{\bmod } = \left[ {\text{NADH}} \right]\left( {\text{t}} \right) \cdot \alpha_{NADH} + A_{extr} $$

with *α*_*NADH*_ being the specific absorbance of NADH. To take into account the sample specific matrix effect (see Fig. 4), the specific absorbance of NADH was determined for every sample following:

8 $$ \alpha_{NADH} = \left( {A_{0} - A_{final} } \right)/[{\text{NADH]}}_{ 0} $$

with *A*_0_ the absorption measured at the start of the assay, *A*_final_ the absorption measured at the end of the assay and [NADH]_0_ the starting concentration of NADH.

**Fig. 5:**
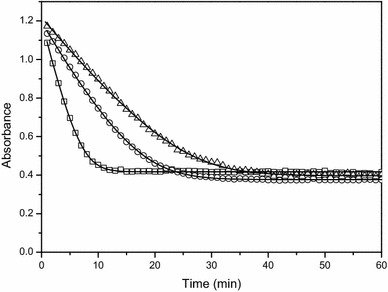
Results of the kinetic model describing the spectrophotometric data of the enzymatic assay to measure alcohol dehydrogenase activity. Results are shown for 3 representative biological apple fruit extracts. Fitted (*line*) and measured (*dots*) values are shown. The estimated ${\text{k}}_{\text{ADH}}^{\prime }$ values were respectively 419 ± 56.5 mol L^−1^ s^−1^ kg^−1^ (*triangle*), 650 ± 2.2 mol L^−1^ s^−1^ kg^−1^ (*circle*) and 1682 ± 56.5 mol L^−1^ s^−1^ kg^−1^ (*square*), all expressed on a total protein weight base

The final model of the ADH assay as defined by Eqs. 6–8 was implemented in OptiPa [16] which was also used to estimate for every sample the unknown model parameters $k_{\text{ADH}}^{\prime }$ and *K*_m,ADH_ by fitting the model to the measured spectrophotometric data. The kinetic model to estimate the enzyme activity of ADH thus takes into account the entire progress curve, as shown in Fig. 5.

### Kinetic model for pyruvate decarboxylase

Since the PDC assay is a coupled assay combining the decarboxylation reaction catalyzed by PDC (Eq. 1) with the indicator reaction catalyzed by ADH (Eq. 2), the kinetic model for the PDC assay has to cover both reactions. The indicator reaction is responsible for generating a measurable spectrophotometric response thanks to the conversion of NADH to NAD^+^. To be able to unequivocally quantify the PDC reaction, the indicator reaction catalyzed by ADH should not become rate limiting. For this reason, commercial ADH was added to the reaction mixture to make sure that acetaldehyde formed in the PDC reaction was swiftly converted into ethanol. As acetaldehyde, as an intermediate substrate, was not assumed to reach saturating levels, first order kinetics was applied to describe the ADH reaction, at least with regard to acetaldehyde. As NADH can be expected to pass through the full concentration range from initial saturating levels to depletion, Michaelis–Menten kinetics was assumed for NADH. Pyruvate levels in the assay were several magnitudes higher than the levels of NADH. As a consequence, the ADH reaction lasts not long enough to act as an indicator for the complete depletion of pyruvate. The part monitored by the assay is therefore only mirroring the initial phase where the PDC reaction operates at its maximum velocity, which can therefore be described using zero order kinetics. The process can thus be defined by the following two equations:

9 $$ \frac{{d[{\text{ACA}}]}}{dt} = k_{PDC} \cdot [{\text{PDC]}}_{0} - \frac{{k_{ADH} \cdot {\text{U}}_{\text{ADH}} \cdot [{\text{ACA}}] \cdot [{\text{NADH}}]}}{{K_{m,NADH} + [{\text{NADH}}]}} $$

10 $$ \frac{{d[{\text{NADH}}]}}{dt} = - \frac{{k_{ADH} \cdot {\text{U}}_{\text{ADH}} \cdot [{\text{ACA}}] \cdot [{\text{NADH}}]}}{{K_{m,NADH} + [{\text{NADH}}]}} $$

where *k*_PDC_ is the rate constant of PDC at saturating pyruvate levels, [PDC] is the concentration of PDC in the reaction mixture, *k*_ADH_ is the rate constant of ADH, K_m,NADH_ the Michaelis–Menten constant with regard to NADH, U_ADH_ the amount of commercial ADH added to the reaction mixture and [ACA], [NADH] are the concentrations of acetaldehyde and NADH in the reaction mixture, respectively.

Like before, the amount of PDC present in the reaction mixture is related to the concentration of enzyme present in the extract ([PDC_extr_]), the volume of the extract (V_extr_), and the total volume of the reaction mixture (V). Hence, Eq. 9 can be written as:

11 $$ \frac{{d[{\text{ACA}}]}}{dt} = k_{PDC} \left( {\frac{{\left[ {{\text{PDC}}_{\text{extr}} } \right] \cdot V_{extr} }}{V}} \right) - \frac{{k_{ADH} \cdot {\text{U}}_{\text{ADH}} \cdot \left[ {\text{ACA}} \right] \cdot \left[ {\text{NADH}} \right]}}{{K_{m,NADH} + [{\text{NADH}}]}} $$

Similar as for the kinetic model of ADH, a composite term $k_{\text{PDC}}^{\prime }$ was introduced which combines the rate constant k_PDC_ and the concentration of PDC in the extract [PDC_extr_] into a single measure of the enzyme activity of PDC as extracted from the sample. Therefore, Eq. 11 was rewritten as follows:

12 $$ \frac{{d\left[ {\text{ACA}} \right]}}{dt} = k_{PDC}^{'} \cdot \left( {\frac{{V_{extr} }}{V}} \right) - \frac{{k_{ADH} \cdot {\text{U}}_{\text{ADH}} \cdot \left[ {\text{ACA}} \right] \cdot \left[ {\text{NADH}} \right]}}{{K_{m,NADH} + [{\text{NADH}}]}} $$

The concentration of NADH at every point in time ([NADH](t)) can be calculated by solving the two coupled differential equations, Eqs. 10 and 12, using [NADH]_0_ and [ACA]_0_ as initial conditions.

According to Fig. 2, not only NADH and the extract, but also pyruvate absorbed UV light at 360 nm. Because the absorbance measured in the enzymatic assay was thus a combination of the absorbance by the changing NADH levels and a constant absorbance (*A*_*final*_) being, on its turn, a combination of a sample specific absorbance of the extract and the constant absorbance by pyruvate, the modelled absorbance *A*_mod_ is given by:

13 $$ A_{\bmod } = \left[ {\text{NADH}} \right] ( {\text{t)}} \cdot \alpha_{NADH} + A_{final} $$

with *α*_*NADH*_ being the specific absorbance of NADH as determined by Eq. 8, and [NADH](t) being the concentration of NADH estimated by the model for every time point.

The final model for the PDC assay as defined by Eqs. 8, 10, 12 and 13 was again implemented in OptiPa and used for estimating the unknown parameter values by fitting the model to the measured spectrophotometric data. The model parameters $k_{\text{PDC}}^{\prime }$ and *K*_m,ADH_ were estimated per sample, but the model parameter *k*_ADH_ was estimated in common over all samples, as the concentration of the enzyme ADH added to the reaction mixture was the same for all of them.

**Fig. 6:**
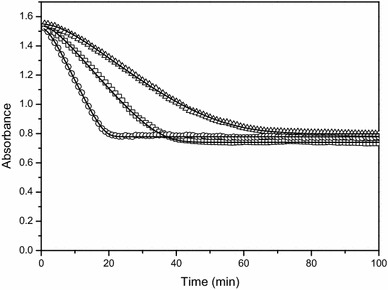
Results of the kinetic model describing the spectrophotometric data of the enzymatic assay to measure pyruvate decarboxylase activity. Results are shown for 3 representative biological apple fruit extracts. Fitted (*line*) and measured (*dots*) values are shown. The estimated ${\text{k}}_{\text{PDC}}^{\prime }$ values were respectively 276 ± 9.5 mol L^−1^ s^−1^ kg^−1^ (*triangle*), 386 ± 5.6 mol L^−1^ s^−1^ kg^−1^ (*square*) and 576 ± 16.9 mol L^−1^ s^−1^ kg^−1^ (*circle*) all expressed on a total protein weight base

The kinetic model to estimate the enzyme activity of PDC takes into account the entire progress curve, as shown in Fig. 6.

### Comparison between linear regression and kinetic modelling approach to estimate enzyme activity

The linear regression approach is generally used to estimate enzyme activity. In the next paragraph a comparison is made between the generally accepted linear regression approach and the kinetic modelling approach to estimate enzyme activity. Advantages of the latter method over the classic method are stressed in the text.

**Fig. 7:**
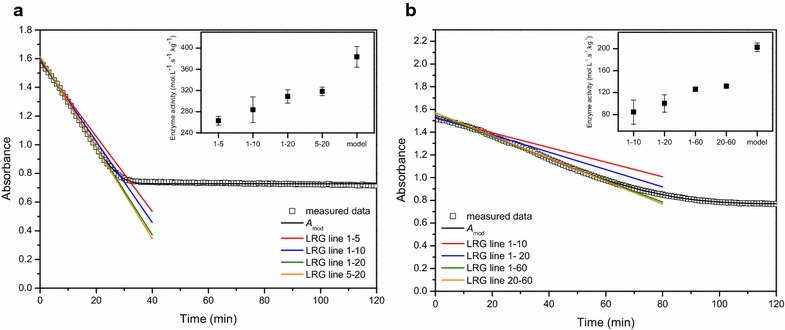
Changes in the final quantification of the slope when applying the linear regression approach. The linear regression approach is indicated by LRG. The results highly depend on whether the progress curve has a short lag phase (**a**) or a long lag phase (**b**) as compared to the linear phase and whether both phases or only the linear part is considered. The *numbers in the legend* indicate the respective start and stop times (in min) used for the linear approximations and the resulting activity calculated. The model outcome (*A*_*mod*_) was based on the full progress curve. The activity calculated through the linear regression approach and the model as well as their 95% confidence interval are indicated on the *graph*

When optimum conditions are applied in the enzyme assay, i.e., substrate and cofactor saturation, standard pH, temperature and ionic strength, the slope of the linear part of the progress curve can be taken as a measure of the maximum enzyme activity. To correctly compare the activity of different enzymes, it is important to always quantify their activity under the optimum working conditions. Like mentioned in the introduction, to experimentally realize substrate saturation, the substrate is required to be present in a 100-fold surplus of the K_m_ value. However, in the case of NADH, to stay within the detection range, the NADH concentration should not exceed 0.2 mM [5]. As a result, it is practically impossible to estimate the theoretical maximum enzyme activity of PDC and ADH using a linear regression approach. Using the kinetic modelling approach the maximum activity of the enzyme can be estimated through extrapolation even if the saturating substrate conditions were not fully realized. The onset of the progress curve of single step reactions, like ADH, approximates linearity (Fig. 1a). With the linear regression approach it is advisable to take a linear part long enough to get reliable results, especially in the presence of remarkable scattering of the data, without extending into the nonlinear part of the progress curve. Using the kinetic modelling approach the whole progress curve is taken into account making this method more robust to measurement noise. It becomes more complex in the case of a coupled reaction, like for PDC, where the progress curve often does not show an initial linear response as a function of time (Fig. 1b). Depending on the size of the lag phase relative to the linear phase, and depending on whether both phases or only the linear part is considered, the final quantification of the slope will change (Fig. 7). As an example, linear approximations were done for two extracts, one with a high (Fig. 7a) and one with a low PDC activity (Fig. 7b). Depending on the sample and the time interval used for the linear approach the obtained values are different. As more time points are used for the linear regression approach, the enzyme activity becomes higher and the 95% confidence interval becomes smaller. If only the linear part of the curve is considered, the enzyme activity calculated is the most reliable. Furthermore, it is important to mention that, in case of the coupled PDC reaction, the enzyme activity estimated by the linear regression approach is a lumped value, encompassing the activity of both PDC and ADH. In contrast, when using the kinetic model the activity of PDC and ADH are estimated separately. This accounts for the completely different values obtained by the model (Fig. 7). The accuracy of the enzyme activity estimated by the model comes from an estimation error and is typically 1.77% but is not affected by the user’s choice of time interval and the fitness of the assumption of linearity.

### Insights given by the model

**Fig. 8:**
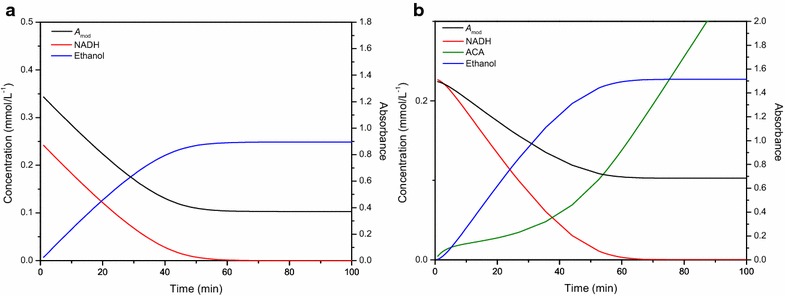
Changes in concentration of all the compounds involved in the reaction. This figure shows the changes in concentration of all the compounds involved in the reaction of alcohol dehydrogenase (**a**) and pyruvate decarboxylase (**b**), simulated by the kinetic models, in relation to the progress curve estimated by the model (*ACA* acetaldehyde)

Figure 8a shows the data simulated by the model for the ADH assay. Once NADH is depleted, the reaction stops and ethanol is no longer produced. In the case of the coupled PDC reaction, the reaction mechanism is more complex.

Since two reactions are involved in the PDC assay, it takes a while before the formation and conversion of the intermediate becomes constant, causing an initial lag phase before reaching the linear phase. As can be seen from the simulated model data (Fig. 8b), only once an initial amount of acetaldehyde is formed the conversion to ethanol by ADH becomes more or less constant, causing the initial lag phase of 5–10 min. While the progress curve seems to be quite linear from 10 min onwards, the slowly increasing level of acetaldehyde is indicating the contrary. With depleting levels of NADH the indicator reaction slows down further, resulting in an ongoing faster accumulation of the intermediate acetaldehyde. The moment NADH is completely finished, the indicator reaction stops and acetaldehyde is no longer converted to ethanol, causing an increase in the concentration of acetaldehyde as the PDC reaction will continue regardless (Fig. 8b).

Of course, one should be aware of the limitations of the kinetic model related to the assumptions applied. As the model was focused towards the reaction period where NADH would still be present, the eventual depletion of pyruvate was not incorporated resulting in an everlasting increase of acetaldehyde. In reality, if the reaction would be allowed to continue, pyruvate would effectively be depleted after 100 h or more. From this it can be concluded that the assumption made was acceptable for the current application.

**Fig. 9:**
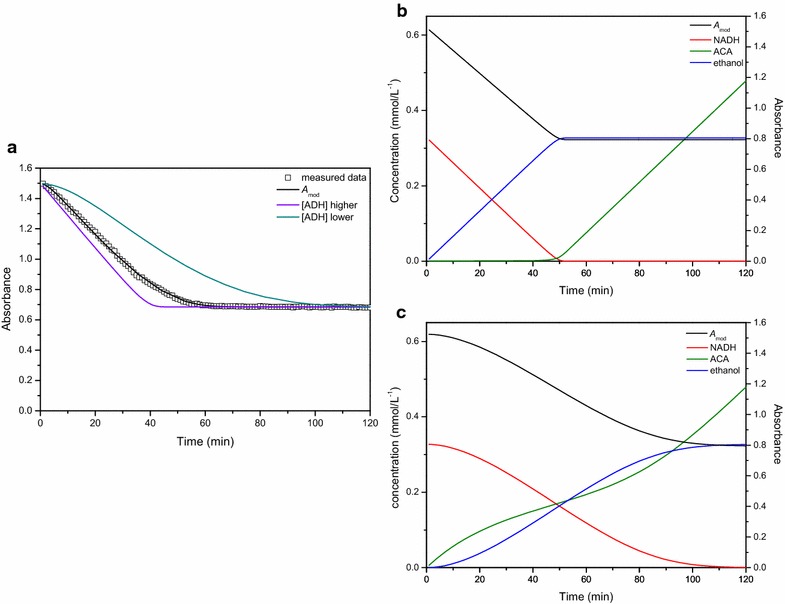
The models can help to optimize the assays by systematically studying the effect of the various design parameters. Changes in the progress curve if more or less alcohol dehydrogenase is added (**a**). Changes in concentration of all the compounds involved in the assay when high amounts (**b**) or low amounts (**c**) of alcohol dehydrogenase (ADH) are added (*ACA* acetaldehyde)

Furthermore, the models can help to optimize the assays by systematically studying the effect of the various design parameters like extract volume, substrate concentration and indicator enzyme amount. For instance, it was hypothesized that the initial lag phase in the PDC assay could be prevented by increasing the amount of ADH, in which case the initial amount of acetaldehyde that needs to accumulate before the conversion to ethanol starts would be less. As shown in Fig. 9a, when the total enzyme activity of ADH is increased by adding more enzyme, the second reaction is no longer rate limiting resulting in a clear initial linear decrease with time as acetaldehyde is converted to ethanol much faster (Fig. 9b). When the amount of ADH added to the reaction mixture is decreased, the opposite effect is observed and the nonlinearity of the curve becomes more pronounced (Fig. 9a). In this case the ADH reaction becomes rate limiting, as indicated by the increasing time needed for acetaldehyde to accumulate before being converted to ethanol.

While the accuracy of the linear regression approach will benefit from an increased ADH level, the kinetic modelling approach can deal with nonlinear results, not necessarily requiring an increased ADH level. As an additional advantage, costs of the assay can be reduced.

## Conclusion

Existing enzyme assays to measure the in vitro specific enzyme activity of PDC and ADH were optimized in order to increase the signal to noise ratio. An elaborated kinetic model based method was proposed to estimate the in vitro specific enzyme activity from the spectrophotometric assay data. The kinetic models developed in this study can accurately estimate the enzyme activity of PDC and ADH in the given assays. In contrast to the linear regression approach, the models can be used to estimate the enzyme activity whether or not linearity is achieved since they take into account the complete progress curve. Using the kinetic modelling approach the maximum activity of the enzyme can be estimated through extrapolation even if the saturating substrate conditions were not fully realized in the enzyme assay, making it possible to compare the obtained results between different experiments and between different labs and publications. The developed models can be applied to routinely analyze continuous spectrophotometric enzyme activity data, but like shown in this paper, they can also be used as a tool to optimize the enzyme assays by systematically studying the effect of the various design parameters. Finally, the use of kinetic models to estimate the in vitro specific enzyme activity can be extended to other enzymes as long as the underlying reaction mechanism is known and the reaction dynamics can be monitored in a continuous way. Furthermore, the differential equations of the developed models can be implemented in any software environment that supports estimation of kinetic parameters on ODE based models, e.g. Matlab (The MathWorks, Inc., Natick, MA, USA), Mathematica (Wolframe, Inc., Boston, MA, USA), Maple (Maplesoft, Inc., Japan) and R (R Foundation for statistical computing, Vienna, Austria), or in online web tools such as ENZO (enzyme kinetics [17].

## Additional files

**Additional file 1.** ADH_model.

**Additional file 2.** PDC_model.

## Abbreviations

ACA: acetaldehyde
ADH: alcohol dehydrogenase
HPLC: high-performance liquid chromatography
MES: 2-(*N*-morpholino)ethanesulfonic acid
ODE: ordinary differential equation
PDC: pyruvate decarboxylase
