# Supplementary material for: Kinetic modelling: an integrated approach to analyze enzyme activity assays
Source: Plant Methods. 2017 Aug 25;13:69. doi: 10.1186/s13007-017-0218-y (PMC5574136; doi:10.1186/s13007-017-0218-y)
Supplement: Supplementary file 1 — Additional file 1. ADH_model. [file 13007_2017_218_MOESM1_ESM.docx]

# ADH_model

%----------------------------------------------------------------------------

% ParamDef: Initialising parameters.

%----------------------------------------------------------------------------

<ParamDef>

%----------------------------------------------------------------------

% Defining constant global parameters.

% These parameters are not available for optimization.

%----------------------------------------------------------------------

cNADH = 2.519; % Absorption coefficient of NADH (OD/(mmol/L))

V = 250; % Total volume of the reaction mixture (µl)

Vextr = 100; % Volume of the extract (µl)

NADH0 = 0.4; % Starting concentration of NADH (mmol/L)

%----------------------------------------------------------------------

% Declaration of model parameters including initial values assigned.

% These parameters are available for optimization

%----------------------------------------------------------------------

<Param> Km_ADH = 1.687;

<Param> K_ADH = 0.0031096;

<Param> A_0 = 1.2;

<Param> A_final = 0.4;

%----------------------------------------------------------------------

</ParamDef>

%----------------------------------------------------------------------------

% StateDef: Assigning initial concentration of NADH and ethanol

%----------------------------------------------------------------------------

<StateDef>

<State> NADH = (A_0-A_final)/cNADH;

<State> ethanol = 0;

</StateDef>

%----------------------------------------------------------------------------

% ModelDef: Defining michaelis menten equation for NADH and ethanol

%----------------------------------------------------------------------------

<ModelDef>

%----------------------------------------------------------------------

Deriv(NADH) = -Km_ADH *(Vextr/V)*NADH/(Km_ADH +NADH);

Deriv(ethanol) = Km_ADH *(Vextr/V)*NADH/(Km_ADH +NADH);

%----------------------------------------------------------------------

</ModelDef>

%----------------------------------------------------------------------------

% TransOut: The output of the model is the modelled absorbance

%----------------------------------------------------------------------------

<TransOut>

%----------------------------------------------------------------------

<Output> Amod = NADH*((A_0-A_final)/NADH0)+A_final;

%----------------------------------------------------------------------

</TransOut>
