# Supplementary material for: Kinetic modelling: an integrated approach to analyze enzyme activity assays
Source: Plant Methods. 2017 Aug 25;13:69. doi: 10.1186/s13007-017-0218-y (PMC5574136; doi:10.1186/s13007-017-0218-y)
Supplement: Supplementary file 2 — Additional file 2. PDC_model. [file 13007_2017_218_MOESM2_ESM.docx]

# PDC_model

%----------------------------------------------------------------------------

% ParamDef: Initialising parameters.

%----------------------------------------------------------------------------

<ParamDef>

%----------------------------------------------------------------------

% Defining constant global parameters.

% These parameters are not available for optimization.

%----------------------------------------------------------------------

cNADH = 2.519; % Absorption coefficient of NADH (OD/(mmol/L))

V = 250; % Total volume of the reaction mixture (µl)

Vextr = 100; % Volume of the extract (µl)

U_ADH =50; % the amount of commercial ADH added to the reaction mixture (Units)

NADH0 = 0.4; % Starting concentration of NADH (mmol/L)

%----------------------------------------------------------------------

% Declaration of model parameters including initial values assigned.

% These parameters are available for optimization

%----------------------------------------------------------------------

<Param> k_PDC = 0.0064931;

<Param> k_ADH = 0.041912;

<Param> A_final = 0.6;

<Param> Km_NADH = 0.04;

%----------------------------------------------------------------------

</ParamDef>

%----------------------------------------------------------------------------

% StateDef: Assigning initial concentration of NADH, Acetaldehyde and ethanol

%----------------------------------------------------------------------------

<StateDef>

<State> NADH = NADH0;

<State> ACA = 0;

<State> ethanol = 0;

</StateDef>

%----------------------------------------------------------------------------

% ModelDef: Defining differential equations for NADH, ACA and ethanol

%----------------------------------------------------------------------------

Deriv(NADH) = -k_ADH*U_ADH*NADH*ACA/(Km_NADH+NADH);

Deriv(ACA) = k_PDC*(Vextr/V)-k_ADH*U_ADH*ACA*NADH/(Km_NADH+NADH);

Deriv(ethanol) = k_ADH*U_ADH*NADH*ACA/(Km_NADH+NADH);

%----------------------------------------------------------------------

</ModelDef>

%----------------------------------------------------------------------------

% TransOut: The output of the model is the modelled absorbance

%----------------------------------------------------------------------------

<TransOut>

<Output> Amod=NADH*((A_0(1)-A_final)/NADH0)+A_final;

</TransOut>
